# Unraveling the diversity and cultural heritage of fruit crops through paleogenomics

**DOI:** 10.1016/j.tig.2024.02.003

**Published:** 2024-05

**Authors:** Meirav Meiri, Guy Bar-Oz

**Affiliations:** 1 The Steinhardt Museum of Natural History and Israel National Center for Biodiversity Studies, Tel Aviv University, Tel Aviv 6997801, Israel; 2 School of Archaeology and Maritime Cultures, University of Haifa, Haifa, 3498837 Mount Carmel, Israel

**Keywords:** ancient DNA, archaeobotany, cultivar, cultural history, genetic diversity, landrace

## Abstract

Abundant and plentiful fruit crops are threatened by the loss of diverse legacy cultivars which are being replaced by a limited set of high-yielding ones. This article delves into the potential of paleogenomics that utilizes ancient DNA analysis to revive lost diversity. By focusing on grapevines, date palms, and tomatoes, recent studies showcase the effectiveness of paleogenomic techniques in identifying and understanding genetic traits crucial for crop resilience, disease resistance, and nutritional value. The approach not only tracks landrace dispersal and introgression but also sheds light on domestication events. In the face of major future environmental challenges, integrating paleogenomics with modern breeding strategies emerges as a promising avenue to significantly bolster fruit crop sustainability.

## Highlights

Paleogenomics provides new ways to study the cultural history and genetic diversity of landrace fruit crops.
Paleogenomics holds the potential to unlock valuable genetic insights from extinct or ancestral fruit-crop cultivars.
This information enables the reconstruction of important chapters in plant domestication and can track their ways of dispersal, introgression, and potential adaptations.
Case studies on grapevines, date palms, and tomatoes, presented in this review highlight the power of paleogenomics in revealing historical aspects.
The examples discussed demonstrate the importance of legacy/heritage cultivars for enhancing food security and agricultural sustainability in different environmental conditions over time.

## Introduction to fruit-crop horticulture

The origins and migration of fruit crops are inextricably woven into the tapestry of human civilization. Fruits have served not only as nourishment but also as symbols of cultural exchange, commerce, and adaptation for millennia. Fruit-crop horticulture was practiced as early as 7000 years ago in the prehistoric Levant, including five founding Mediterranean crops: olives (*Olea europea*), the common fig (*Ficus carica*), grapevines (*Vitis vinifera*), date palms (*Phoenix dactylifera*), and pomegranates (*Punica granatum*) [1., 2., 3.]. A second pivotal wave of fruit domestication occurred in the first millennium CE, during the Roman, Byzantine, and Early Islamic periods, when interconnected societies significantly increased the transcontinental exchange of plants beyond their centers of origin, and when many varieties of propagated crops were crossed and bred to create locally adapted **landraces** (see Glossary) throughout Eurasia. At this time, fruit crops that were domesticated in central Asia, including apples (*Malus domestica*), pistachios (*Pistacia vera*), and mulberries (*Morus* sp.), as well as peaches (*Prunus persica*) in China and several varieties of citrus (*Citrus* sp.) in southeast Asia, then dispersed and spread across the ancient trade routes. Longstanding human engagements along geo-historical pathways are well documented both historically [4., 5., 6., 7.] and archaeobotanically [8., 9., 10., 11., 12., 13., 14.].

The rich and diverse cultural history of fruit crops is reflected in the thousands of autochthonous heritage **cultivars** mentioned in historic records. Landrace fruit crops are typically adapted to a local environment and traditional farming systems. Consequently, these landraces have distinct genetic identities that have been shaped by extensive cultural histories that developed during the process of selection. Their contributions go beyond serving as repositories of global cultural diversity, identity, and sustainable agricultural practices to fulfilling roles of key economic and nutritional significance. High genetic diversity would have enabled such crops to thrive in adverse environmental conditions and to develop strains resistant to diseases and pests, allowing their cultivators to achieve yield stability [15., 16., 17., 18., 19.]. Nevertheless, many of these cultural heritage landrace fruit crops have either become extinct or remain unidentified. This reduction in genetic diversity – sometimes termed **genetic erosion** – has primarily resulted from the selection of a limited number of high-yielding or visually appealing cultivars [18,20]. Both **genetic loss** and cultural loss have significantly accelerated over the past 150 years with the development of industrialized agriculture and a shift towards modern cultivars and monoculture farming that emphasizes a few dominant strains selected by the demands of the global market [18]. Genetic erosion in fruit crops can be seen, for example, in the cultivated Cavendish banana. The widespread cloning of this variety is by tissue culture, which led to a lack of genetic diversity, resulting in a monoculture that is highly vulnerable to diseases and pests. This poses a significant risk to global banana production similar to the devastating effects of Panama disease on the Gros Michel variety in the past [21].

The restoration of genetic diversity in culturally and economically significant legacy cultivars is crucial to mitigate genetic loss and enhance food security and agricultural sustainability [22]. They may contain valuable genes for traits such as disease resistance, drought tolerance, nutritional content, and flavor profiles. By identifying and reviving these landraces, we can tap into a broader genetic pool to improve crop resilience and productivity. These have evolved through human selection for local adaptation [22., 23., 24.]. Strategies to achieve this in fruit crops include collecting and conserving diverse germplasms, breeding for resistance and adaptability, and *in situ* conservation of landrace fruit crops [23,24]. Molecular techniques such as marker-assisted selection and genetic engineering can also play a pivotal role in revitalizing fruit-crop diversity [23].

## Paleogenomics in archaeobotany

Paleogenomics can also be used to unlock and study the historic genetic diversity and cultural heritage of fruit crops [22]. This emerging field involves the reconstruction and analysis of ancestral genomes by analyzing ancient DNA extracted from archaeobotanical remains [19,22]. Paleogenomics has transformed our understanding of the evolutionary traces of humans and other organisms by reconstructing chapters in the genetic histories of species [3,25., 26., 27., 28., 29.] (Box 1).

Box 1 **Advancements in, and the potential of, paleogenomic research**

Paleogenomics is the study of ancient DNA extracted from preserved biological materials. In many respects it is a revolutionary field that has significantly expanded our understanding of human history, evolution, and environmental adaptation. The retrieval and analysis of ancient DNA from archaeological and paleontological remains has even offered insights into the genetic makeup of extinct and ancient populations [49,99,100], and ancient DNA data can help to address an array of questions in anthropology, evolutionary biology, and the environmental and archaeological sciences.

Such data have, for example, been used to study archaic human groups such as Denisovans and Neanderthals [101,102], to unravel human migration patterns [103,104], and to shed light on domestication processes [105]. Paleogenomics has revolutionized our comprehension of the historical origins and global dissemination of significant infectious diseases [106], contributed to understanding the microbial composition of humanity [107], and to the discovery of epigenetic markers [40]. The knowledge gained from such studies will guide the exploration of ancient holobiomes, which hold the potential to reveal insights into societal, dietary, and environmental transformations, as well as their consequences for individual and population health [49].

Although it holds much potential, paleogenomics is challenged by the inherent instability of DNA molecules because they are susceptible to significant postmortem degradation and environmental contamination in warm and humid environments [41,108]. Efforts have been made to enhance extraction techniques, concentrating on short DNA fragments and specific skeletal elements that yield high amounts of endogenous DNA [109,110], and to develop minimally invasive methodologies [111]. The advent of next-generation sequencing (NGS) technologies has driven the field of ancient DNA research into the realm of paleogenomics by enabling the generation of genome-wide data from limited DNA samples, while progress in library-based reconstruction methods and targeted enrichment techniques has expanded the capacity to obtain sequences from specific genomic regions [50., 51., 52., 53., 54., 55.,^112^,^113^].

Alt-text: Box 1

Studying the genomes of ancient fruit crops allows researchers to assess the genetic diversity present in ancient populations [22,26,30,31]. It also provides insights into domestication processes, revealing the complexity underlying selection, additive functional alleles, and gene flows from wild populations [3,22,32]. This information enhances our understanding of the interactions between humans and plants in ancient agricultural societies [30., 31., 32.]. Many studies of domestication have been carried out only on modern DNA, but employing selective sweeps to pinpoint 'domestication genes' using such DNA can miss earlier selection events – such as those linked to initial domestication – and encounter the limitations of population assumptions [33,34].

Paleogenomics encapsulates an extensive chronicle of adaptations to dynamic conditions, thereby offering insights into how plants navigated and acclimated to novel environments over time. Unraveling the historical contexts of local adaptations opens avenues through which to apply this knowledge to contemporary crops [30,31]. Studies are now beginning to unveil instances of potential adaptations in ancestral populations that are notably absent from present-day crops [35,36]. Pertinently, most of the fruit crops can reproduce through vegetative propagation as well as by sexual reproduction. Vegetative propagation enables genetic diversity to be conserved, and lost ancient cultivars can still be present today, perhaps as feral varieties; it is therefore possible to use paleogenomics to help to reintroduce lost genetic diversity into modern varieties for improved resilience [3,32,37].

**Figure 1:**
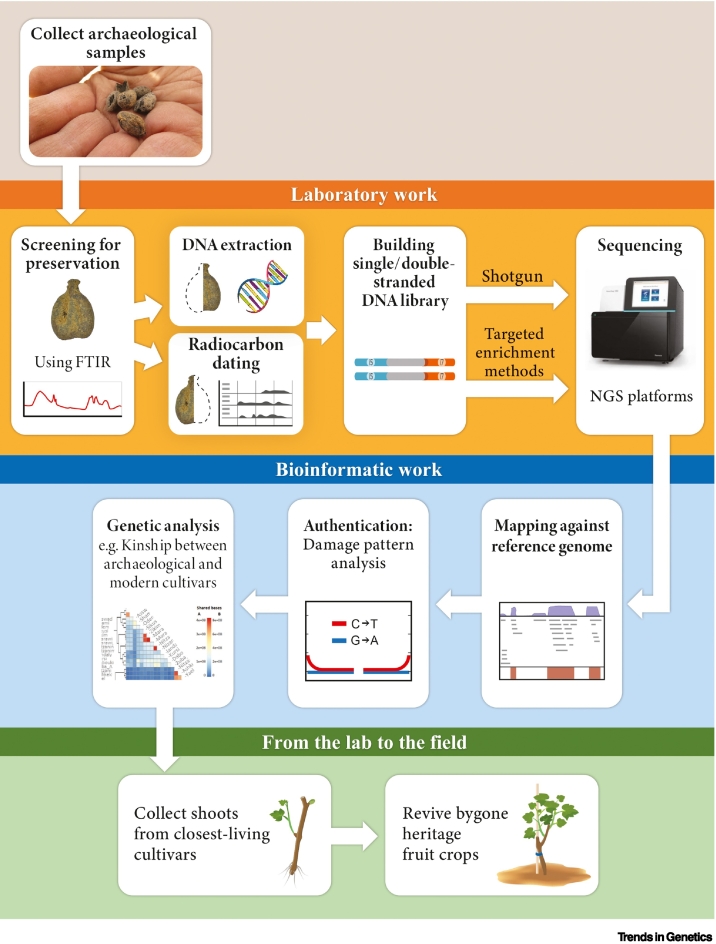
The main methological steps for ancient DNA work: Laboratory work: FTIR is carried out to screen the samples. The best preserved samples are splitted into two; one half of a sample going for ^14^C analysis and the other for DNA extraction in a clean lab, and to the preparation of single-stranded (ss)DNA and/or double-stranded (ds)DNA libraries, which are then sequenced through shotgun or using targeted enrichment methods. (B) Analyses include alignment against reference genomes and assessment of authenticity and postmortem damage, and then various genetic analyses. From the lab to the field: collecting shoots from the closest-living cultivars and planting them to revive bygone heritage crops. Abbreviations: FTIR, Fourier transform infrared; NGS, next-generation sequencing.

Botanical remains can be found in paleontological, archaeological, museum, and herbarium collections. Source materials encompass a range of constituents, including seeds, fruits, wood, pollen, and charred plant remnants [38]. Desiccated and waterlogged constituents commonly have high rates of DNA preservation, whereas charred seeds, despite being the most abundant, usually have poor DNA preservation due to nucleotide damage, short DNA fragments, low endogenous DNA content, and the potential for contamination [39]. Even with the integration of next-generation sequencing (NGS) technologies and targeted enrichment strategies, the efficacy of DNA retrieval from charred remains is limited [40,41]. Fourier transform infrared (FTIR) spectroscopy can be used to evaluate seed preservation conditions and select the best-preserved specimens [29], and, whenever feasible, seeds can be split into two halves: one for radiocarbon (^14^C) dating and the other for DNA extraction (Figure 1). This approach ensures the correlation of DNA sequences with dating information.

To counter challenges posed by the presence of polysaccharides and polyphenols in plant tissues, DNA extraction methods use a CTAB (cetyltrimethylammonium bromide) protocol, and occasionally add PTB (*N*-phenacylthiazolium bromide) to digestion buffers [42]. Some modified methodologies have been also refined to retain ultrashort DNA molecules [43,44]. Genetic libraries have also been tailored to accommodate degraded DNA, including the development of single-tube double-stranded DNA libraries to minimize losses during purification steps [45], and single-stranded DNA libraries to capture both ds and ss components within a sample [46,47]. Enrichment methods have been applied across various species to amplify the yields of specific genetic loci [48] (Figure 1).

DNA sequencing through NGS techniques generates vast datasets, necessitating sophisticated bioinformatic tools for comprehensive analysis. This process encompasses the processing of raw data sequencing; alignment against reference genomes and sequence databases, and assessment of authenticity and error rates, including of miscoding lesions that result from postmortem damage [49]. A diverse array of bioinformatic tools and pipelines are available that are tailored to specific research objectives. These tools facilitate the exploration of genetic affinities among individuals, the determination of kinship relationships, and the elucidation of population ancestry [49] (Figure 1).

**Figure 2:**
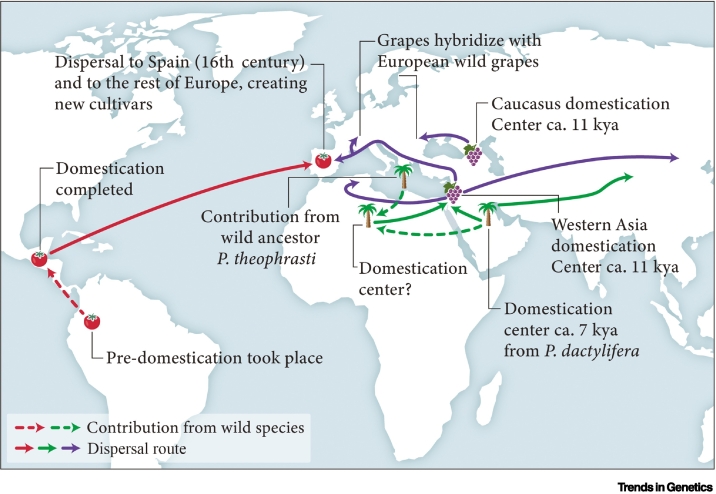
Domestication centers and routes of dispersal for the three case studies as described in the main text. Abbreviations: kya, thousand years ago; *P. dactylifera, Phoenix dactylifera; P. theophrasti, Phoenix theophrasti.*

To date, most paleogenomic research on ancient crops has focused on annual plants such as cereals, legumes, sunflowers, and cotton to examine the pace and targeted selection processes of domestication (maize [50., 51., 52.], sorghum [53], barley [54], emmer [55], sunflower [56], cotton [57]), but little work has focused on fruit crops (e.g., a few studies in the Cucurbitacea family [58., 59., 60.]). To emphasize the potential of such research, we review three recent case studies on the genetic diversity of clonally propagated grapevines (*V. vinifera*), the domestication and **introgression** of the date palm (*P. dactylifera*), and the genetic diversity and evolution of the tomato (*Solanum lycopersicum*) (Figure 2).

## Grapevines

Grapevines are one of the most economically and culturally important fruit crops worldwide. Since their domestication over 6000 years ago in southwest Asia [1,61,62], grapes have primarily been grown for wine production, and archaeological and historical evidence indicates that wine has been an important part of culture ever since. Viticulture (grape-growing) and viniculture (winemaking) have evolved many times and in many places since grapevine domestication [63]. This rich history is reflected by the diversity of wine regions that have produced >6000 legacy cultivars within their individual *terroirs* [64,65]. The >3500 analyses of contemporary grapevine accessions reported by Dong *et al.* [62] revealed parallel domestication in the Near East and the Caucasus. Grapevine dispersal probably accompanied human migration, for example, from the Caucasus into the Carpathian Basin via the northern Black Sea, and from the Near East to Eurasia and North Africa (Figure 2). Dong *et al.* [62] also revealed insights into selection for berry palatability, hermaphroditism, muscat flavor, and skin color, and their work complements other milestone explorations into the cultural history and genetic diversity of the grape [66., 67., 68.].

The *Vitis* genotyping array has a set of 10 000 genetic single-nucleotide polymorphisms (SNPs), each carefully selected to allow highly precise cultivar identification and genetic relationships between closely related grapevines and a variety of characteristics, including berry color, grape acidity, and time of ripening [69]. Genetic markers have also proved useful for paleogenomic studies and have led to a series of advances in reconstructing ancient grape DNA [70,71] and the taxonomic cultural history of viticulture. Ancient DNA extracted from grape pips at French archaeological sites has shown that Western European grapevine cultivars have kinship relationships with those of mediaeval and Roman times [27]. Remarkably, one 900-year-old specimen was identified as the genetic clone of Sauvignon Blanc, a popular modern cultivar, offering evidence for centuries of uninterrupted clonal propagation. The spread of viticulture to the western Mediterranean during the Bronze Age [mid-second millennium BCE (before common era)] involved hybridization between imported domestic cultivars, most likely arriving from the Aegean, and local wild grapevines [72].

Further insights into historical viticulture traditions come from the Negev Desert of southern Israel, where recent paleogenomic studies have led to the discovery of cultivars that were bred ~1100 years ago and were sufficiently resilient to grow in the *terroir* of a hyper-arid environment (aridity index ≤ 0.10; mean annual rainfall 80–100 mm/year [29]). These cultivars are a prime example of sustainable and sophisticated dryland viticulture in the first millennium CE (common era) [73]. The genetic signature of one Negev grape pip dated to the ninth century CE was found to be the antecedent of a modern Greek cultivar and has been linked to several popular historic wines that were traded across the Byzantine Empire. Another Negev pip dated to eighth century CE was probably from a white grape – the earliest white grape identified to date [29]. The genetic diversity observed in these two seeds reinforces the hypothesis that vineyards were established using a range of crop cultivars. Such an approach would have extended the harvest period, as grapes of different cultivars ripen gradually throughout the season. The similarity between Byzantine Negev grapes and modern cultivars provides strong evidence for stable vegetative propagation and cultivar continuity over 1000 years.

## Date palms

Dates are a major fruit crop in the Middle East and North Africa [74]. Date palms play a crucial role in fostering economic, ecological, and cultural value in microclimates within oasis ecosystems, and facilitate sustainable agriculture in arid zones. Their sweet, nutrient-rich fruits are a vital nutritional element, while other parts of the plant find everyday utility, such as the stipe (trunk) in construction and the leaves in basketry [74,75].

There are currently >3000 named date cultivars worldwide [76], but the production of dates has shifted from traditional cultivation in diverse agrosystems to intensive monoculture systems [77]. For instance, >70% of date palm orchards in Israel are of the Medjool variety, and this number is steadily rising [78]. This transformation to a monoculture can potentially expose the entire crop to threats from novel pathogens, pests, and climatic changes, and thus exemplifies the need to restore historical data and preserve genetic diversity.

The domesticated date palm is thought to have originated in the Middle East during the fourth millennium BCE. Dates were subsequently spread throughout North Africa by the Roman period [79,80]. Genetic studies suggest that relict populations of the wild progenitor of date palms (*P. dactylifera*) persist in the Hajar Mountains of Oman in the Arabian Peninsula, and that this may have been the center of date domestication [81,82] from which date palms were likely spread both eastward and westward. However, African date palms display greater diversity and distinctiveness than Middle Eastern cultivars and challenge the conventional notion of a single domestication center in the Near East [83,84] (Figure 2). Dates were extensively cultivated in Egypt from at least the mid-second millennium BCE, and further west in the Maghreb by at least the first millennium BCE [83]. The paleogenomic study of a ~2100-year-old date palm leaf from Saqqara, Egypt, reveals the geneflow into North African date palms from two *Phoenix* species: *Phoenix theophrasti*, that is found in a limited distribution area extending between Crete and other Aegean islands [85,86], and *Phoenix sylvestris* in Central Asia [87]. Genetic data supports introgression from *P. theophrasti* into the domesticated date palm ~3000 years ago, leading to North African date palms containing as much as 18%of the wild Cretan genome [81,83,84]. Whether this introgression mirrors selection preferences for 'wild alleles' or is indicative of disparities in demographic history and hybridization remains a subject of ongoing research [84].

Another study focused on 2000-year-old germinated seeds from the Judean Desert (Israel) and indicate genetic intermixing between Middle Eastern (eastern) and North African (western) date palm gene pools [88,89]. Seeds from the fourth to first centuries BCE were more closely related to modern eastern date cultivars, while those from the second century BCE to the second century CE exhibit increasing genetic affinities with present-day North African date palms. These findings align with samples from the southern Levant, a land bridge between Asia and Africa, where it appears that local farmers were keen on preserving genetic diversity in their crops by cross-breeding with foreign male plants [88]. The change in the genetic makeup in the Judean Desert over a few centuries probably results from the influence of market demands, dictated by changing institutional constraints and the dictates of imperial powers [89].

## Tomatoes

The domesticated tomato holds immense importance for the culinary, nutritional, and economic aspects of human life. Tomato domestication manifests a complex path which probably began in the Andean region of Ecuador and Peru, and was completed in Mesoamerica [90]. It is generally depicted as a 'two-step' process in which the wild species, *Solanum pimpinellifolium*, was domesticated in South America to give rise to S. *lycopersicum cerasiforme* (cherry tomato), which later gave rise to *S. lycopersicum lycopersicum* (large-fruited tomato, now the most popular cultivated tomato) in Mesoamerica [91,92] (Figure 2). However, a recent study has shown that the cherry tomatoes originated in Ecuador ~80 000 years ago, long before human groups began to domesticate plants, and probably started as a wild species [93]. In the 16th century CE, as part of the Columbian Exchange, tomatoes were introduced to Europe and to other parts of the world [91] (Figure 2). These human-induced migration processes and associated selection processes reduced the genetic diversity of tomatoes and resulted in the near fixation of a large proportion of the tomato genome, including a dramatic increase in fruit size [92].

To better understand the history of tomato loci selection, the whole genome was sequenced from two specimens taken from herbarium cards from 18th and 19th century Italy [28]. One of the samples, dated to ~1750 CE, showed high genetic similarity to the genome of tomato landraces obtained from the Campania region, the same area as the herbarium collection. The second sample, dated to 1890 CE, had elongated fruits and was genetically distant from cultivated varieties, suggesting that elongated tomato cultivars might have originated from a cross between a landrace and a wild ancestor before they reached Italy. This study highlights the importance of appreciating domestication and migration processes when understanding the genetic history of cultivars, and thus maintaining genetic diversity.

Further research explored well-preserved tomato specimens from a 16th century herbarium, called the ‘En Tibi’ herbarium, which probably originated in Bologna, Italy, at around 1558 [94,95]. It has been suggested that these old tomato cultivars could help to revive ancient resistance to pests and diseases, and thus contribute to the development of new cultivars with the 'original' taste. In their review, Andel *et al.* [96] screened early 16th century tomatoes mentioned in descriptions or visible herbarium specimens, including the ‘En Tibi’ herbarium, to show that different landraces of tomatoes were introduced to Europe from Mesoamerica very soon after its discovery. They noticed great variety in flower and fruit shapes, sizes, and colors, indicating that the earliest tomatoes in Europe came in a much wider variety of than had previously been thought. Ancient nuclear and plastid genomes were sequenced from one ‘En Tibi’ herbarium specimen [97], and the resulting genomic data showed that this was a fully domesticated tomato, genetically close to three Mexican landraces, and the specimen therefore probably derived from a cultivar from around the Gulf of Mexico. The specimen was also more heterozygous than all recently collected accessions from Mesoamerica, which means that it was less inbred or domesticated than present-day tomatoes. The study on ‘En Tibi’ herbarium specimen provides a snapshot of the dynamics of tomato domestication and cultivation through time and space. It also highlights the significance of paleogenomics in identifying ancient and traditional tomato landraces, particularly in Central and South America where genetic similarities are prevalent.

## Future perspectives

Paleogenomics holds the potential to unlock valuable genetic insights from extinct or ancestral fruit-crop cultivars. The case studies presented in this review illuminate the continuity of several cultivars through time [27,28], and reveal the challenges presented by the distribution of samples across wide geographic areas in which potentially thousands of autochthonous landraces were developed over time through continuous cross-species hybridization and introgression. Complex webs of breeding and selection also result from ease of hybridization and different modes of propagation practices, further complicating the analysis. However, this information is crucial for understanding how crops evolved and adapted to different environmental conditions over time. Ancient gene sequences cannot revive lost variation. However, beneficial genetic traits from ancient varieties can be identified and reintroduced, such as drought-related genes or temperature-adapted genes, to enhance the resilience and adaptability of modern crops [30., 31., 32., 33.]. To date, paleogenomics in fruit crops has only dealt with phenotypic traits such as berry color in grapevines [29] or flesh color in watermelon where the bitter pulp was also studied [58]. By contrast, in other crops such as maize traits related to adaptability are well studied [35]. To reach the full potential of paleogenomics when investigating the evolutionary history of fruit crops, it is crucial to expand the research and to include more ancient samples and more fruit taxa with wider taxonomic, geographic, and spatial distributions (see Outstanding questions).

**Figure 3:**
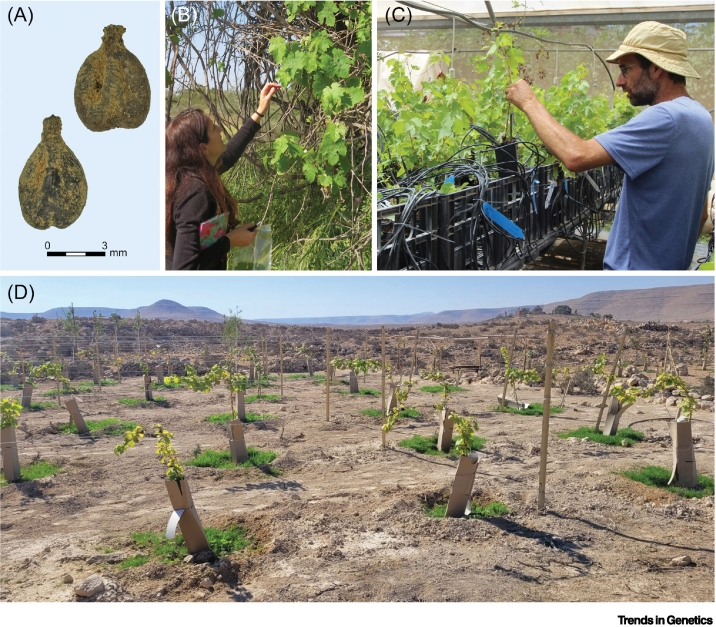
(A) Archaeobotanical grape pips. (B) Collecting samples of feral grapevines for DNA fingerprinting. (C) Cultivating grapevines from rotted cuttings sourced from the Western Negev in a heritage tree nursery. (D) Establishing an inaugural heritage vineyard in the Negev Highlands.

The International Union for Conservation of Nature (IUCN) has highlighted the risks of monocultures to food production, and acknowledge the threats facing the diversity of indigenous endemic crops as a result of habitat destruction and the loss of landrace genomes [98]. Paleogenomic studies of archaeobotanical fruit crops can indicate that native cultivars only underwent limited sexual reproduction and, unlike seed-propagated annual crops, were dispersed mainly through cloning, as shown for the grapevine [27., 28., 29.]. Moreover, many historical landraces are still extant, and historical fruit-crop landraces may survive in the wild as relicts, as feral plants, or are kept in botanical collections that preserve local fruit crops. Paleogenomics can therefore be used to trace historical hybrids, and can bolster the resilience, adaptability, and nutritional quality of fruit crops worldwide to ensure their viability in changing environments and enhance global food security (Box 2) (Figure 3) (see Outstanding questions).

Box 2 **Negev heritage vineyard concept**

The revival of heritage grapes that share a paleogenetic footprint with existing feral grapevines serves as a proof of concept for planting ancient fruit-tree cultivars that were traditionally propagated through tree cloning and grafting (see Figure 3 in the main text). Analyses of kinship relationships between ancient pips and modern cultivars provide compelling evidence for stable intergenerational vegetative propagation spanning more than a millennium. Autochthonic Negev grapevine cultivars possess intrinsic value in their ability to withstand dryland habitats which can significantly impact on traditional wine-growing areas, and can mitigate the effects of desertification.

For the project, we propagated cuttings from the associated feral cultivars and closely monitored them at a dedicated nursery. In September 2023, the newly propagated cultivars were planted in an inaugural 'heritage vineyard' situated near ancient viticultural remnants at Avdat, the archaeological site at which the grape seeds were discovered. These newly planted archaic grapevines establish a direct link with the historical wine culture of the region [114], and have generated interest from stakeholders keen on integrating our 'from near extinction to market distinction' methodology into the global wine market. The vineyard also empowers research-based educational and tourism initiatives intended to promote awareness of heritage horticulture and helps to turn the tide on losses of landrace species.

Alt-text: Box 2

## Concluding remarks

Fruit crops have long been, and continue to be, crucial for the nutritional viability of the world. Given the increasing recognition that food monocultures have a detrimental effect on this viability, it is correspondingly evident that alternatives should be sought. Legacy cultivars, and particularly fruit-crop cultivars, are a valuable resource for maintaining global nutritional viability. Paleogenomic research is uniquely positioned to identify and trace the developmental lineage of such alternatives, while offering multiple opportunities for ongoing research.

Outstanding questions How can paleogenomic studies be optimized to identify specific genes in ancient fruit crops that are associated with adaptations to abiotic (e.g., temperature fluctuations, drought, salinity), biotic (e.g., pests and disease), and anthropogenic (e.g., cultivation management and land use) stressors?

What are the challenges and opportunities to bridge the gap between paleogenomic discoveries and their practical implementation in breeding programs, particularly in terms of improving fruit cultivar yields and enhancing crop performance?

In what ways can paleogenomics contribute to the documentation and preservation of traditional knowledge of autochthonous landrace fruit crops, thereby ensuring that their distinct identity is integrated into future agricultural strategies?

How can paleogenomic research on fruit crops be translated into educational initiatives, museums, or cultural institutions to raise awareness about the rich cultural and geographic histories embedded in these crops and promote sustainable agricultural practices?

Alt-text: Outstanding questions

## Glossary

**Cultivar**: a plant variety that has been produced during cultivation by selective breeding.
**Genetic erosion**: loss of genomic variation and the accumulation of detrimental mutations within a population, which can result in reduced fitness levels.
**Genetic loss**: a reduction or eradication of specific genetic traits or information within a population over time. This might occur through various mechanisms including genetic drift, natural selection, restricted gene flow, inbreeding, and small population size, or other evolutionary forces. The reduction in genetic diversity may impact on the adaptability of a population to changing environmental conditions.
**Introgression**: the transfer of genetic material from one species into the gene pool of another through repeated backcrossing.
**Landrace**: a crop variety or population managed by farmers through cultivation, selection, and diffusion, which is typically adapted to a local area and to traditional farming systems, has a recognizable identity and geographic origin, and is often genetically heterogeneous [115].
